# Poem: Waiting for the barbarian Corona viruses

**DOI:** 10.11613/BM.2021.010401

**Published:** 2020-12-15

**Authors:** Eleftherios P. Diamandis

**Affiliations:** 1 Department of Pathology and Laboratory Medicine, Mount Sinai Hospital, Toronto, Canada; 2 Lunenfeld-Tanenbaum Research Institute, Mount Sinai Hospital, Toronto, Canada; 3 Department of Laboratory Medicine and Pathobiology, University of Toronto, Toronto, Canada; 4 Department of Clinical Biochemistry, University Health Network, Toronto, Ontario, Canada

## Note from the author

My “poem” represents a serious effort to modify the famous poem by legendary Greek poet Constantine Cavafy entitled “Waiting for the barbarians”, which was written in the city of Alexandria, Egypt, in 1898. The meaning/message between the original poem and the one that I adapted is exactly the opposite. In Cavafy’s original poem, there is a burning desire of the people who live in a city to have their problems solved by an exogenous power, even if this power is “the barbarians”. In other words, those in some kind of trouble, call for other people to come and solve the problems. In my version, which I related to the COVID-19 infections, to make it timely, the citizens of the city are pleading for the bad occurrence (in this case the COVID-19 virus disease) to magically disappear as soon as possible; and they hope that this will solve their problem. Unfortunately, as my version posits, the problem came and it will likely be permanent. So, the citizens have to find a way to live with the problem. Needless to say, “the problem” can be anything from old age, to cancer, dementia, divorces, financial difficulties *etc.*

A video version of the poem can be viewed at the link: https://www.youtube.com/watch?v=enC8I88XRl8.

## Waiting for the barbarian Corona viruses

What are we waiting for, assembled in our houses?
The Corona viruses are due to leave today.
Why isn’t anything going on in the senate?
Why are the senators sitting there without legislating?
Because the Corona viruses are due to leave today
What’s the point of senators making laws now?
Once the Corona viruses leave, isolation measures are not necessary anymore.
Why did our emperor get up so early?
And why is he sitting enthroned at the city’s main gate,
In state, wearing the crown?
Because the Corona viruses are due to leave today
And the emperor’s waiting to see the crown of their leader.
He’s even got a scroll to give him
Loaded with names of thousands of dead people.
Why have our two consuls and praetors come out today?
Wearing their embroidered, their scarlet togas?
Why have they put on bracelets with so many amethysts?
Rings sparkling with magnificent emeralds?
Why are they carrying elegant canes
Beautifully worked in silver and gold?
Because the Corona viruses are due to leave today
and things like that run the corona viruses away.
Why don’t our distinguished orators turn up as usual?
To make their speeches, say what they have to say?
Because the Corona viruses are due to leave today
And Corona viruses are getting bored with nonsense crap.
Why this sudden bewilderment, this confusion?
(How serious people’s faces have become.)
Why are the streets and squares emptying so rapidly?
Everyone going home lost in thought?
Because night has fallen and the Corona viruses did not leave
And some of our men just in from the border say
The Corona viruses will never leave.
Now what’s going to happen to us with the Corona viruses on our back?
Their magical departure was a kind of a solution.

